# βγ G-proteins, but not regulators of G-protein signaling 4, modulate opioid-induced respiratory rate depression

**DOI:** 10.3389/fphys.2023.1043581

**Published:** 2023-04-06

**Authors:** Jamil Danaf, Carolina da Silveira Scarpellini, Gaspard Montandon

**Affiliations:** ^1^ St. Michael’s Hospital, Unity Health Toronto, Toronto, ON, Canada; ^2^ Department of Medicine, University of Toronto, Toronto, ON, Canada

**Keywords:** opioid-induced respiratory depression, medulla, G-protein, preBotzinger complex, opioid receptors, regulators of G-protein-signaling, breathing

## Abstract

Opioid medications are the mainstay of pain management but present substantial side-effects such as respiratory depression which can be lethal with overdose. Most opioid drugs, such as fentanyl, act on opioid receptors such as the G-protein-coupled µ-opioid receptors (MOR). G-protein-coupled receptors activate pertussis toxin-sensitive G-proteins to inhibit neuronal activity. Binding of opioid ligands to MOR and subsequent activation G proteins βγ is modulated by regulator of G-protein signaling (RGS). The roles of G-proteins βγ and RGS in MOR-mediated inhibition of the respiratory network are not known. Using rodent models to pharmacologically modulate G-protein signaling, we aim to determine the roles of βγ G-proteins and RGS4. We showed that inhibition of βγ G-proteins using gallein perfused in the brainstem circuits regulating respiratory depression by opioid drugs results in complete reversal of respiratory depression. Blocking of RGS4 using CCG55014 did not change the respiratory depression induced by MOR activation despite co-expression of RGS4 and MORs in the brainstem. Our results suggest that neuronal inhibition by opioid drugs is mediated by G-proteins, but not by RGS4, which supports the concept that βγ G-proteins could be molecular targets to develop opioid overdose antidotes without the risks of re-narcotization often found with highly potent opioid drugs. On the other hand, RGS4 mediates opioid analgesia, but not respiratory depression, and RGS4 may be molecular targets to develop pain therapies without respiratory liability.

## Introduction

Opioid medications are widely used in pain management (Gutstein et al., 2001; Montandon et al., 2016a), but are highly addictive (Wilkerson et al., 2016) and can present the severe side-effect of respiratory depression (Dahan et al., 2010; Montandon et al., 2016b; Nagappa et al., 2017) that can be lethal with overdose (Jones et al., 2013; Gomes and Juurlink, 2016). The current epidemic of opioid overdoses claimed over 80,000 lives in the United States in 2020 and is responsible for millions of emergency visits every year, therefore causing a severe social, health and financial burden (Florence et al., 2016). Respiratory depression by opioids is due to misuse of street drugs like heroin, but equally due to prescription pain killers (Gutstein et al., 2001; Gomes and Juurlink, 2016). No opioid pain therapies are available with limited respiratory depression (van der Schier et al., 2014). The opioid antagonist naloxone or Narcan blocks the effects of opioids, but also blocks analgesia so cannot be used as an adjunct treatment to prevent respiratory depression. In addition, naloxone induces severe withdrawal symptoms in opioid-dependent patients (Purssell et al., 2021) therefore limiting its use. The risks of re-narcotization while using naloxone as an antidote is considerable because highly potent opioid drugs such as fentanyl and its analogs (Pergolizzi, 2021) have longer half-lives than naloxone. The development of potent analgesic therapies with reduced respiratory depression as well as new overdose antidotes has been hindered by the lack of understanding on the mechanisms of action of opioids (Montandon and Slutsky, 2019), and the absence of molecular targets and drug candidates that could provide safe, effective analgesia.

**FIGURE 1:**
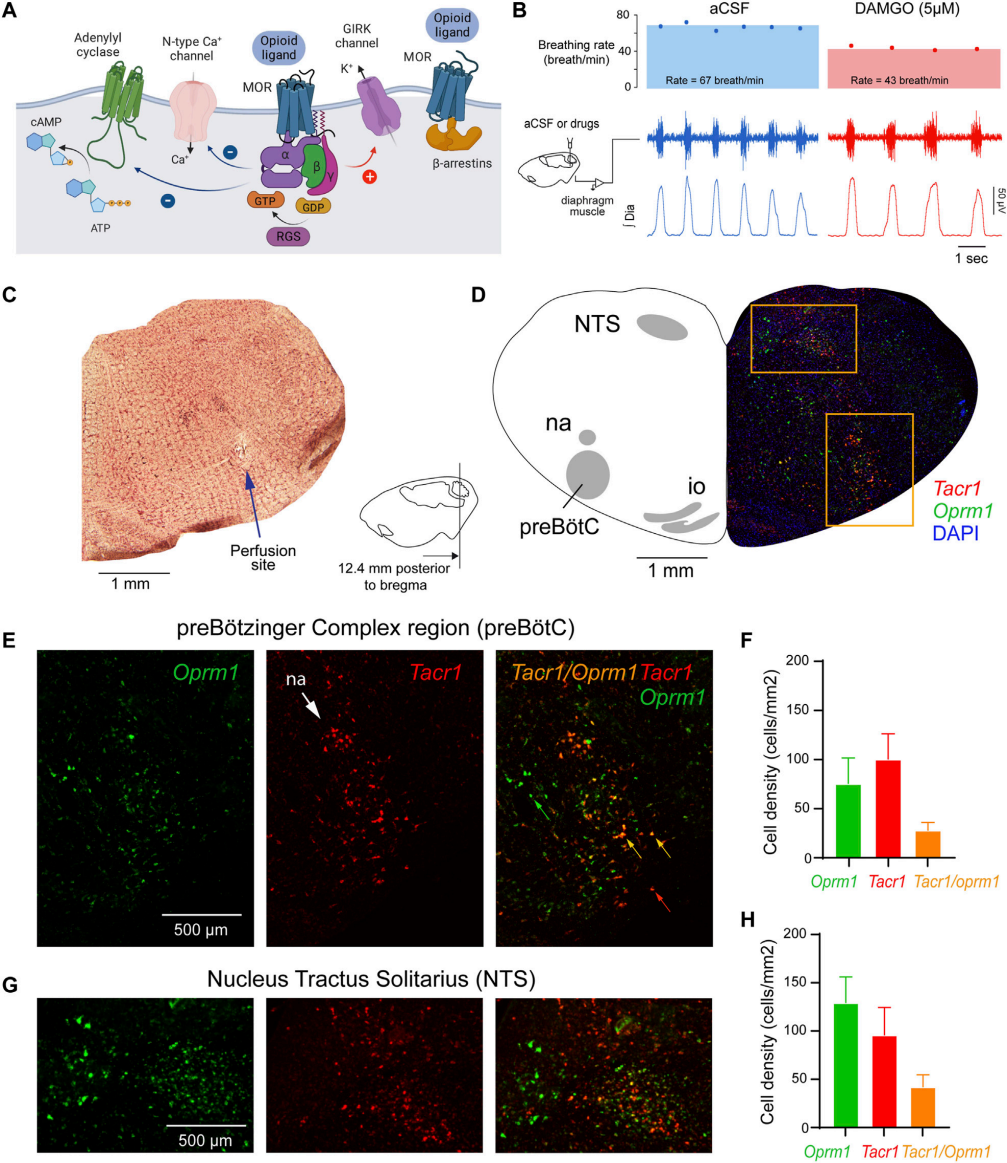
Neural mechanisms of neuronal inhibition by MORs and expression of *Oprm1* and *Tacr1* (the gene encoding neurokinin-1 receptors) in the medulla. **(A)** Neuronal inhibition by MORs is regulated by G-protein-signaling including α, *ß* and γ proteins and regulators of G-protein signaling. **(B)** Microperfusion of the MOR agonist DAMGO directly into the preBötC depressed respiratory rate in anesthetized rats. **(C)** The location of the microperfusion site was identified with histological lesion made by the microdialysis probe shaft. Note that the probe extends beyond the guide by 0.5 mm. **(D)** The preBötC and the nucleus tractus solitarius (NTS) expressed *Oprm1* (the gene coding for MORs) and *Tacr1* (the gene coding for tachykinin-1 or neurokinin-1 receptors) mRNAs **(E, F)** The density of *Oprm1* and *Tacr1* were assessed in the preBötC and the NTS **(G, H).** MOR, µ-opioid receptor. GTP, guanine trisphosphate. GDP, guanine diphosphate. RGS, regulators of G-protein-signaling. GIRK, G-protein-activated inwardly rectifying potassium channels. Dia, diaphragm muscle. aCSF, artificial cerebrospinal fluid. Mean data are presented as means ± SEM. Panel a was created using Biorender.com.

Most opioid drugs, such as oxycodone and fentanyl, activate µ-opioid receptors (MORs) and inhibit neuronal activity through three canonical pathways (Figure 1A). MOR activation opens G-protein-gated inwardly rectifying potassium (GIRK) channel (Marker et al., 2004; McCoy and Hepler, 2009), inhibit N-type calcium channels, and/or inhibit adenylyl cyclase/cAMP (Gutstein et al., 2001). G-protein-coupled receptors activate pertussis toxin-sensitive G-proteins, and K^+^ currents are elicited due to contacts of βγ proteins (Gβγ) and GIRK channels (McCoy and Hepler, 2009). MORs also modulate voltage-gated calcium channels through binding of Gβγ to regulate their calcium influx (Weiss and Zamponi, 2020). Binding of opioid drugs to MORs also inhibits the enzyme adenylyl cyclase, which, in turn, reduces the conversion of ATP into cyclic adenosine monophosphate (Gutstein et al., 2001). In addition to these three canonical pathways, it has been suggested that *ß*-arrestin recruitment mediates respiratory depression by opioid ligands but has limited role in opioid analgesia (Bohn et al., 1999; Raehal et al., 2005). However, this concept has been recently challenged. It was originally asserted that analgesia by opioid ligands is mediated by G-protein signalling and GIRK channels, while respiratory depression would be mediated by recruitment of *ß*-arrestin (Bohn et al., 1999; Raehal et al., 2005). However, in mice with a non-functional G protein-receptor kinase 2, which prevents recruitment of *ß*-arrestin by MORs, the dose of fentanyl needed to reduce breathing rate was signiflcantly lower than wild-type mice (Kliewer et al., 2019), suggesting that *ß*-arrestin does not mediate respiratory depression by opioid drugs. Consistent with a role of G-protein signalling, instead of *ß*-arrestin recruitment, we showed previously that GIRK channels mediate respiratory depression by opioid drugs (Montandon et al., 2016c). In knockout mice lacking the GIRK2 subunit, a subunit constituting most neuronal GIRK channels, respiratory depression by the opioid drug fentanyl was substantially reduced compared to wild-type mice (Montandon et al., 2016c). Here, we propose that inhibition of respiratory circuits by MORs is mediated by G-protein signaling.

Binding of opioid ligands to their cognate MORs induces a conformational change of the intracellular G-protein Gα subunit, thus facilitating the switch from the Gα subunit GDP-bound inactive state to its GTP-bound active state and dissociation from the Gβγ subunit complex (Pasternak and Pan, 2011). The Gα and Gβγ complexes then diffuse through the cytoplasm to various target molecules in order to activate their secondary messenger pathways (Pasternak and Pan, 2011), including calcium and GIRK channels. The roles of Gβγ subunit and the specific Gβ and Gγ isoforms in regulating opioid-induced respiratory depression are not known. G-proteins involved in MOR inhibition can be modulated by various regulators of G-protein signaling (RGS) (De Vries and Gist Farquhar, 1999) that bind to the Gα subunit (De Vries et al., 2000). With binding of RGS proteins, the Gα_i/o_ and Gβγ subunits are no longer able to carry out the signal initiated by ligands binding to the MOR, such as activation of GIRK channels by Gβγ (Doupnik et al., 1997). Various RGS proteins have been implicated in opioid signal pathways with the RGS4 highly expressed in the amygdala, locus coeruleus, nucleus accumbens, and throughout the spinal cord (Gold et al., 1997). The role of RGS proteins in regulating MOR inhibition of respiratory circuits is not known.

We propose to test the roles of two key-mechanisms of G-protein signaling in opioid-induced respiratory depression. We aim to elucidate the role of Gβγ subunits and RGS proteins found in the neural circuits mediating respiratory depression by opioids. We propose to modulate the activity of Gβγ and RGS proteins in the preBötzinger Complex (preBötC), a brainstem site important for opioid-induced respiratory depression (Montandon et al., 2011) and for the generation of rhythmic breathing (Smith et al., 1991). Using pharmacological interventions directly on neural circuits regulating breathing, we probe the roles of these mechanisms in opioid-induced respiratory depression, without affecting other neural circuits, which may indirectly affect respiratory depression by opioids. This study supports the concept that respiratory depression is mediated by MOR activation, G-protein signaling and activation of downstream effectors such as GIRK channels or calcium channels (Montandon et al., 2016b).

## Materials and methods

All procedures were performed in accordance with the recommendations of the Canadian Council on Animal Care and were approved by the St. Michael’s Hospital Animal Care Committee. All studies were performed in adult male Wistar rats with body weight between 200 and 300 g from Charles River Laboratories. Male rats were exclusively used because female rats have been shown to have differential skull and brain anatomies (Hughes et al., 1978).

### Anesthetized experiments in rats

To determine the role of G-protein signaling on respiratory rate depression by MOR agonists, we used reverse microdialysis to continuously perfuse agonists, antagonists, or blockers into the preBötC of male Wistar rats while recording respiratory muscle activity as described previously (Montandon et al., 2011). Rats were anesthetized with isoflurane (3%–4%) mixed with 50% oxygen and then tracheostomized. Isoflurane was then maintained at 1.5%–2.5%. Body temperature was monitored using a rectal probe (TC-1000 Temperature Controller, CWE Inc., Ardmore, PA, United States), and kept at 37°C. Two stainless steel needle electrodes were inserted into the tongue to measure genioglossus muscle activity. Two electrodes made of stainless-steel braided wire were inserted inside the abdomen under the right costal diaphragm to measure diaphragm muscle activity. Genioglossus muscle activity was closely monitored, as previous studies show it decreases by about 30% when the probe is lowered near the preBötC (Montandon et al., 2011; Montandon et al., 2016c), likely due to the probe interfering with premotor neurons that project to the hypoglossal motor nucleus. Diaphragm muscle activity, genioglossus muscle activity, and breathing rate were recorded throughout. The raw signals were amplified (1,000x), filtered (band-pass filter 10-1,000Hz), and recorded using the Spike2 software (Cambridge Electronic Design, Cambridge, UK). The rat was placed on a stereotaxic frame (model SAS-4100, ASI Instruments Inc., Warren, MI, United States) in the prone position and ear bars and a mouthpiece were used to stabilize the skull. A microdialysis probe (CX-I-12-01, 200 µm diameter, 1 mm length of diffusing membrane, Eicom United States, San Diego, CA, United States) was inserted in the region of the preBötC. The stereotaxic coordinates were determined according to previous studies, which used a combination of coordinates from the Rat Atlas and neurokinin-1 receptor (NK-1R) expression in the ventrolateral medulla to locate the preBötC (Montandon et al., 2011; Montandon and Horner, 2013; Montandon et al., 2016b). The probe was unilaterally inserted into the brainstem 12.2 mm posterior, 10.5 mm ventral, and 2.0 mm lateral to the bregma. Drugs were continuously perfused at 3 μL/min. The probe was inserted close to the preBötC to allow drugs perfuse to the preBötC in the allotted time frame. To ensure that the probe was located in the vicinity of the preBötC, we determined whether microperfusion of DAMGO (5 µM) decreased respiratory rate by 10% in less than 30 min 30 min was used as a strict cut-off interval based upon previous studies (Montandon et al., 2011; Montandon et al., 2016) and preliminary data. Although increasing the time interval would potentially lead to more successful experiments, it would also cause the drugs to diffuse to areas further than the preBötC, and thus decrease the spatial specificity of this approach.

### Microperfusion of drugs into the medulla

To manipulate MORs, RGS4, and Gβγ, combinations of drugs were perfused into the preBötC of anesthetized rats. All drugs were purchased from Tocris Bioscience. The MOR agonist DAMGO was microperfused at a concentration of 5 µM which was significant to decrease respiratory rate by 10% or more but not high enough to completely stop breathing (Montandon et al., 2011). CCG 50014 (20 µM) is a selective RGS4 inhibitor. Naloxone (20 µM) is an MOR antagonist. Gallein (1 and 5 mM) is a Gβγ inhibitor. As gallein is insoluble in water, dimethyl sulfoxide (DMSO) was used as a vehicle to dissolve it in artificial cerebrospinal fluid (aCSF). Considering that the final concentration of DMSO was 5% due to low solubility of gallein, we performed negative controls where solutions of aCSF, DMSO (5%) and gallein (5 mM) were microperfused in the preBötC of adult rats. aCSF was perfused for all baseline recordings. The composition of aCSF is as follows: 125 mM NaCl, 3 mM KCl, 1 mM KH_2_PO_4_, 2 mM CaCl_2_, 1 mM MgSO_4_, 25 mM NaHCO_3_, and 30 mM glucose. The pH of the solution was adjusted down to 7.4 by bubbling CO_2_ into the aCSF.

### Determination of Probe Site Location

Histology was performed post-mortem to determine the location of the probe. After rats were overdosed with 5% isoflurane, they were transcardially perfused with saline solution followed by formalin (10%) to fixate tissue. The brain was removed and stored in formalin (10%), then transferred to 30% sucrose solution and stored in the refrigerator. After 24 h in sucrose, brainstem tissue was frozen in a cryostat (Leica Biosystems, Weltzar, Germany) at −20°, cut coronally into 50 µm thick sections, and mounted onto slides. Hematoxylin and eosin were used to stain tissue sections. Slides were visualized under ×4 magnification of an Olympus upright B×50 light microscope (Olympus, Tokyo, Japan). Using anterior-posterior, dorsal-ventral, and medial-lateral coordinates in standard brain maps (Paxinos and Watson, 2014) as well as anatomical markers such as the NA, facial nucleus, and inferior olive, the stereotaxic coordinates of the probe sites and the preBötC were determined (Montandon et al., 2011; Montandon and Horner, 2013). Animals with probes placed more than 1 mm away from the center of the preBötC, as determined by post-mortem histological analysis, were not considered for analysis.

### *In vivo* experimental procedures

We first determined whether Gβγ inhibition could modulate respiratory rate depression by DAMGO. For baseline recordings, we microperfused aCSF for 30 min into the preBötC, followed by microperfusion of DAMGO for 30 min, and subsequent microperfusion of gallein (1 mM) for 40 min concurrently with 5 µM DAMGO. In a separate set of experiments, we investigated if a higher dose of gallein (5 mM) could fully reverse respiratory rate depression. At the end of each experiment, naloxone (20 µM) was perfused to determine whether it is possible to fully reverse respiratory rate depression by DAMGO. As gallein was dissolved in DMSO, DMSO was also added to the naloxone + DAMGO drug combination as vehicle condition. In the second set of experiments, we determined the role of RGS4 in regulating respiratory inhibition by DAMGO.

We first microperfused aCSF for baseline recordings, followed by either the RGS4 inhbitor CCG 50014 in treatment animals or aCSF in controls, each for 40 min. We then microperfused 5 µM DAMGO with CCG 50014 or 5 µM DAMGO alone for 30 min. In a separate set of experiments, we performed the same protocol with higher concentration of CCG 50014. In this second set of experiments, we then followed each experiment with administration of 20 µM naloxone concurrent with DAMGO in controls, and in DAMGO and CCG 50014 in treatment group. For each drug condition, respiratory rate and diaphragm muscle amplitude were reported as average values over the final 10 min of the drug perfusion condition. Due to variability in baseline respiratory rate in-between experiments, we normalized data for each experiment. Respiratory rates and diaphragm muscle amplitudes were normalized as percentage of baseline activity.

### Correlation Maps

To determine whether drugs were perfused in areas of the brainstem regulating breathing, we established the relationship between the position of the microdialysis probe and drug effects on respiratory activity as previously demonstrated (Montandon et al., 2011; Montandon and Horner, 2013). The assumption is that perfusion of drugs close to the preBötC, which is central to the generation of respiratory rhythm and respiratory depression, would have fast effects on respiratory variable, while perfusion at longer distances from the preBötC would have limited effects. We related the distance of the probe perfusion site from the center of the preBötC with the latency of DAMGO to depress respiratory rate. The probe perfusion site was determined using post-mortem histological analysis. The center of the preBötC was determined using a combination of anatomical markers such as the facial nucleus, inferior olive, nucleus ambiguus from post-mortem histological analysis and stereotaxic coordinates (Paxinos and Watson, 2014), as well as expression of *Tacr1* mRNAs (for the gene coding for NK-1R). Drug latency was defined as the time for DAMGO administration to decrease respiratory rate by 10%. Using this concept, we generated correlation maps where red indicates areas where DAMGO had fast effects on respiratory rate and blue slow effects. The reasoning behind constructing these maps is that, if a specific area in the brainstem is responsible for mediating opioids effects on respiratory rate, then the time needed for the drug to diffuse through the brainstem tissue to the site of interest and gradually decrease respiratory rate would be dependent on the distance of the probe from the site of interest.

### *In situ* hybridization

To determine the expression of *Oprm1*, *Tacr1* and *Rgs4*, we used RNA-based *in situ* hybridization assay (RNAscope ACD Bio, Newark, CA, United States). *Oprm1* is the gene coding for MORs, *Tacr1* for tachynin-1 receptors or NK-1R, and *Rgs4* for regulators of G-protein signaling 4. Rats were euthanized with a 5% isoflurane overdose and transcardially perfused with phosphate buffered saline, followed by formalin, and brains were placed in formalin, 10%, 20%, and then 30% sucrose solutions overnight. Brainstem tissue was frozen in a cryostat at −20° (Leica Biosystems, Weltzar, Germany) and cut coronally into 25 µm thick sections. Target retrieval reagent (ACD Bio, Newark, CA, United States) was applied directly to slides. Protease solution (ACD Bio, Newark, CA, United States) was added onto slides, followed by application of the *Oprm1* and *Rgs4* mRNA probe. Slides were then incubated in multiplex fluorescent v2 AMP1 and AMP2 solutions (ACD Bio, Newark, CA, United States) to amplify signal. TSA Cy3 fluorescent dye solution (Perkin Elmer, Waltham, MA, United States) was added for *Oprm1* signal, and Cy5 fluorescent dye was added for *Rgs4* or *Tacr1* signals. DAPI counterstain was added to sections, slides were mounted with fluorosave (Millipore Sigma, Burlington, MA, United States), coverslipped, and dried overnight. Sections were scanned using Zeiss Axioscanner (Carl Zeiss AG, Oberkochen, Germany) to visualize staining patterns. Various landmarks, including the nucleus ambiguus, inferior olive, and XII nucleus, along with molecular markers, such as *Tacr1* expression, were used to locate the preBötC. Borders of the preBötC were determined according to rat brain with stereotaxic coordinates for Wistar rats (Paxinos and Watson, 2014). Negative controls were used to set a threshold of fluorescent expression. Negative control probes targeted the bacterial *Dapb* gene, which is not expressed in rat neural tissue. Positive control probes included *Polr2a*, *Ppib*, and *Ubc* genes, which are common housekeeping genes. Images were acquired with Zen software (Zeiss), were assembled for presentation using Adobe Photoshop and Adobe Illustrator (Creative Suite 15). Labelled neurons were counted and aligned for averaging according to defined anatomical landmarks. Average cell counts obtained from one section from each mouse were used as a single data point for subsequent analysis. We considered *Tacr1*-positive cells inside a square of 800 × 800 µm positioned below the nucleus ambiguus which includes all preBötC cells (Supplementary Figure S1, S6). As a control structure, we looked at the NTS region by counting labelled cells in a rectangle of 1,200 × 800 µm including the NTS. To identify these areas in tissue sections, the Mouse Brain in Stereotaxic Coordinates (third Edition, Paxinos and Franklin) was consulted, and anatomical markers included the NTS, the NA, the facial nucleus, the hypoglossal nucleus, the cerebellum and the inferior olive were used. We used the manufacturer’s *Guide for RNAscope Data Analysis* (Advanced Cell Diagnostics, Newark, California, United States) to count and distinguish cell mRNA expression from background expression. Cells were considered to express mRNA if 4 or more dots (one dot represents one mRNA molecule) and/or 1 or more clusters of dots overlapped with or were adjacent to a DAPI-stained cell nucleus. Using this approach, we quantified the density of labelled cells per mm^2^. The fractional expression of *Oprm1* or *Tacr1*-positive cells was then quantified for each animal.

### Statistical Analysis

Data were presented in figures as mean values ±standard error of the means. N values for each group were indicated in the figure captions. For studies with gallein, one-way repeated-measure ANOVAs were used, with the repeated factor being conditions (aCSF or drugs). For studies with CCG 50014, two-way repeated-measure ANOVAs were used, where the repeated factor was condition (baseline, DAMGO, naloxone) and the non-repeated factor intervention (control vs. RGS4 inhibitor). Normality was tested with the Shapiro-Wilk test and homogeneity of variances were tested using the equal variance test or Brown-Forsythe test. When tests were statistically significant with *p < 0.05*, Holm-Sidak post-hoc tests were performed to uncover the differences in the two factors. When data was non-normal, one- or two-way repeated ANOVAs on ranks were performed. All statistical analyses and figures were performed and constructed using SigmaPlot 14 (Systat Software Inc., San Jose, CA, United States). Graphs were then further edited with Adobe Illustrator (Adobe Inc., San Jose, CA, Un9ited States) for improved styling and to create multi-panel figures. MOR signaling diagrams were created using BioRender (BioRender, Toronto, ON, Canada).

## Results

Activation of MORs in the preBötC decreases respiratory rate in anesthetized rats (Figures 1B, C) (Montandon et al., 2011). To confirm that DAMGO activates MORs located in the preBötC region, we determined the density of MOR-expressing cells in adult rats (Montandon and Horner, 2014). Here, we first determined whether MORs are expressed in the preBötC using *in situ* hybridization (Shi et al., 2021). We looked at the co-expression of *Oprm1* (the gene coding for MORs) and *Tacr1* mRNAs (the gene coding for tachykinin-1 or neurokinin-1 receptors, NK1-R, a receptor known to be highly expressed in the preBötC of adult rodents (Stornetta et al., 2003a)). *Oprm1* RNA was expressed in the preBötC region with a density of 79.7 cells/mm^2^ (Figures 1D–F). *Tacr1* was expressed in this region with a density of 101.6 *Tacr1*-positive cells/mm^2^. Cells positive for *Oprm1* and *Tacr1* had a density of 27.6 cells/mm^2^. Importantly, 73.6% of *Tacr1*-positive cells co-expressed *Oprm1*, suggesting that a substantial part of *Tacr1*-positive cells are *Oprm1*-positive. Another well-defined region of the medulla, the NTS, showed high expression of *Oprm1* and *Tacr1* cells **(** Figures 1G,H). The density of *Oprm1*-positive cells was of 129.2 cells/mm^2^ and density in the NTS was substantially higher than in the preBötC (*p = 0.002,* n = 3). Interestingly, the density of *Tacr1*-positive cells was also high at 86.1 cells/mm^2^ in the NTS, and 21.5% of *Tacr1* cells co-expressed *oprm1* RNA. In conclusion, these results confirmed a relatively high density of *Tacr1*-positive cells in the preBötC region with 73.6% of these cells co-expressing *Oprm1*, which corresponds to the preBötC region where DAMGO was perfused.

**FIGURE 2:**
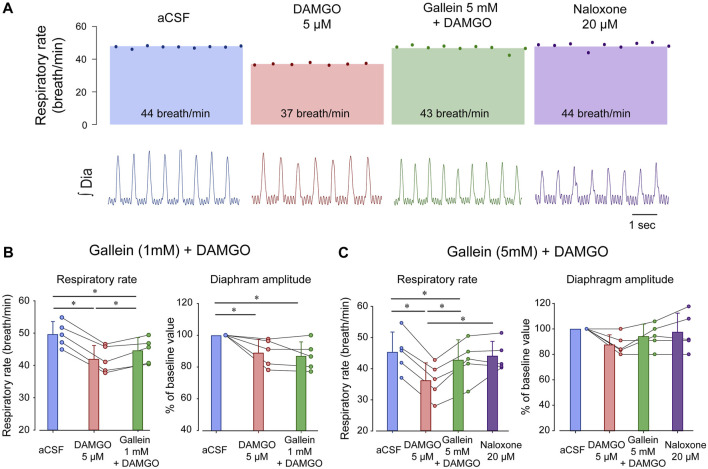
Inhibition of βγ proteins reverses respiratory rate depression by the MOR agonist DAMGO. **(A)** DAMGO reduced respiratory rate by about 15.5% when compared to aCSF or vehicle, and this reduction was reversed by gallein at 5 mM, but only partially at 1 mM. Naloxone did not increase respiratory rate beyond the aCSF or gallein values. **(B)** DAMGO reduced respiratory rate compared to aCSF and gallein (1 mM) partially reversed respiratory rate. Although diaphragm amplitude was reduced by DAMGO, this reduction was not reversed by gallein. **(C)** DAMGO significantly depressed respiratory rate, an effect completely reversed by gallein at 5 mM. No effects were observed in diaphragm amplitude. aCSF, artificial cerebrospinal fluid. MOR, µ-opioid receptors. Bars show means ± SEM. Each circle represents an individual experiment. * indicate means significantly different with *p < 0.05*.

To determine the role of βγ proteins in regulating respiratory rate depression due to MOR activation in the preBötC, we microperfused DAMGO or a combination of DAMGO and the βγ protein inhibitor gallein (Liu et al., 2018) in anesthetized rats (Montandon et al., 2011). DAMGO (5 µM) depressed respiratory rate by 15.5% (*p < 0.001*, n = 5, Figure 2A), and gallein (1 mM) combined with DAMGO partially reversed respiratory rate depression (*p < 0.001*, n = 5, Figure 2A). DAMGO also decreased diaphragm amplitude by 11.0% (*p = 0.039*, n = 5), a decrease not reversed by gallein (*p = 1.000*, n = 5). To determine whether a higher concentration of gallein would completely reverse respiratory rate depression by DAMGO, we increased gallein concentration to 5 mM. At this concentration, gallein reversed substantially respiratory rate depression induced by DAMGO (*p = 0.001*, n = 5, Figures 2A, C). To determine whether gallein has the same ability to reverse respiratory rate as the MOR antagonist naloxone, we perfused naloxone at 20 µM. Naloxone did not further change respiratory rate demonstrating that gallein had the same capacity to reverse respiratory rate depression as naloxone (*p = 0.100*). Diaphragm amplitude was not changed by DAMGO, gallein nor naloxone (*p = 0.075*). In conclusion, the respiratory rate depression by DAMGO was reversed by the βγ protein blocker gallein which supports the hypothesis that βγ proteins mediate MOR-inhibition of respiratory circuits.

**FIGURE 3:**
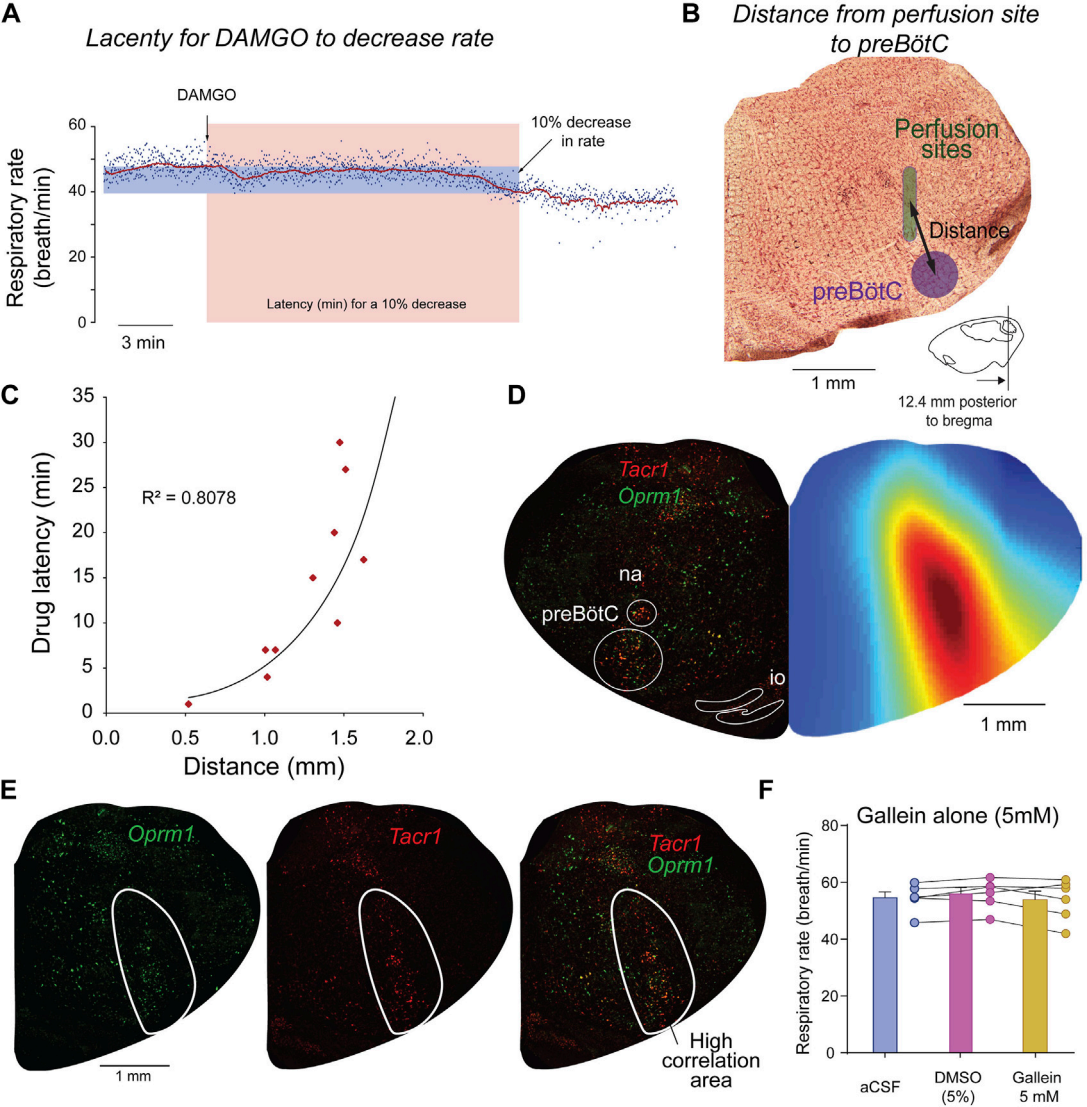
Site of action of DAMGO and gallein overlaps with *Oprm1* and *Tacr1* expressions. To determine the brainstem region where DAMGO is acting, we correlated the latency for DAMGO to decrease respiratory rate with the distances from the preBötC to the microperfusion sites. The latency in minutes for a 10% decrease from baseline respiratory rate was calculated for each experiment **(A)**. For each corresponding experiment, the distance from the microperfusion site (indicated in green) to the center of the preBötC was calculated **(B)**. The coordinates for the preBötC are 12.4 mm posterior, 6 mm ventral, and 2.0 lateral to Bregma. A significant exponential correlation was found between drug latencies and distances (R^2^ = 0.8078, *p < 0.001*, n = 10 experiments) **(C)**. To determine whether other sites in the brainstem may mediate some of the respiratory depression induced by DAMGO, we generated a color map of the brainstem section where each pixel (every 50 µm) corresponds to a coefficient of correlation (blue equals low correlation, whereas red high correlation). For each set of coordinates in the section, the correlation coefficient was calculated using latencies and the distances from the microperfusion sites to the coordinates of the corresponding pixel. This correlation map shows that the area of the brainstem where the correlations are the highest corresponds to the region of the preBötC **(D)**. The highly significant part of the correlation map (R^2^ > 0.5) overlapped with cells labelled with *Oprm1* and *Tacr1* **(E)**. Control experiments were performed with microperfusion in the preBötC of aCSF, artificial spinal fluid, DMSO, dimethyl sulfoxide (5%) or gallein (5 mM) alone. See Methods for additional information on correlation maps as well as previous studies using this approach (Montandon et al., 2011; Montandon et al., 2016a).

One of the limitations of using pharmacological interventions in live rodents is that drugs can diffuse beyond their intended targets. To confirm that perfusion of DAMGO and gallein was performed in the preBötC region, we identified the locations of the perfusion sites for each experiment. We then correlated the latency for DAMGO to depress respiratory rate (Figure 3A) with the distances from the microperfusion sites to the preBötC (Figure 3B). The reasoning behind this approach is that microperfusion performed close to the site of action of the drug would quickly reduce respiratory rate, whereas a microperfusion site located away from the site of action would show longer latency (Montandon et al., 2011). We found a significant correlation between drug latencies and distances (Figure 3C). Therefore, microperfusion close to the preBötC elicited a faster decrease in respiratory rate than microperfusion away from this region (Figure 3C **),** which suggests that DAMGO acts directly at the preBötC and does not act on other regions of the brainstem regulating respiratory rhythm. To confirm that this is only true for the preBötC region and not for other regions of the medulla, we calculated correlation coefficients between latencies and distances from microperfusion sites for all possible coordinates on the medullary section. We then plotted the correlation coefficients as color pixels in red for highly correlated and blue for low correlation. Each pixel was plotted every 50 µm. The region of the medulla showing the highest correlations corresponds to the region were drugs had the fasted effects of breathing rate (Figure 3D). In addition, the region indicated in red corresponded to a region with high density of *Tacr1* and *Oprm1* cells which is consistent with the preBötC region (Montandon et al., 2016a) and an opioid-sensitive site.

To determine that the effects observed were not due to the vehicle used to dissolve gallein and to determine whether gallein alone may increase respiratory rate when perfused in the preBötC, we performed a series of control experiments. Microperfusion in the preBötC of the vehicle DMSO (5%) did not change significantly respiratory rate compared to aCSF (*p = 0.254*, n = 5, Figure 3F). Perfusion of gallein alone (5 mM) did not significantly change respiratory rate compared to aCSF (*p = 0.6500*) or DMSO (*0.2953*, Figure 3F) despite perfusion in the preBötC confirmed by histology (Supplementary Figure S3). In conclusion, inhibition of βγ proteins by gallein reversed the effects of DAMGO on respiratory rate, an effect not observed when gallein was microperfused alone.

**FIGURE 4:**
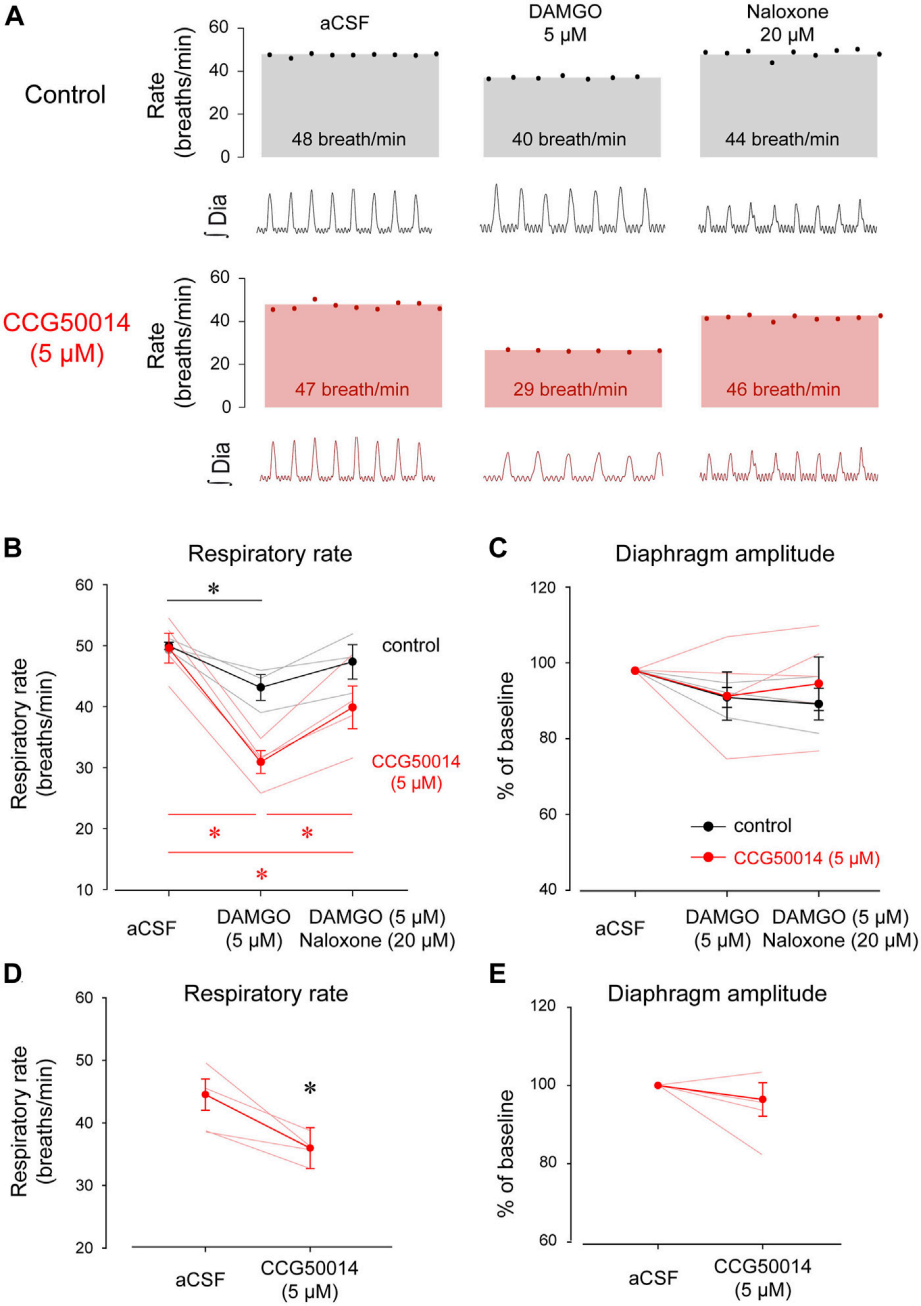
Inhibition of RGS4 has limited impact on respiratory rate depression by DAMGO. **(A)** Inhibition of RGS4 potentiates MOR inhibition by the ligand DAMGO. **(B)** Pharmacological inhibition of RGS4 by CCG50014 significantly accentuated respiratory depression by DAMGO perfused in the preBötC compared to DAMGO alone. **(C)** The combination of DAMGO + CCG50014 (5 µM) induced a stronger respiratory rate depression than DAMGO alone (5 µM). Naloxone partially reversed respiratory rate depression by DAMGO and CCG50014. **(D)** Diaphragm amplitude was not significantly reduced by DAMGO with or without CCG50014. To determine whether RGS4 inhibition alone affects respiratory rate, we microperfused CCG50014 to the preBötC. CCG50014 (5 µM) decreased significantly respiratory rate **(E)** but not diaphragm amplitude **(F)**. aCSF, artificial cerebrospinal fluid. MOR, µ-opioid receptors. RGS, regulators of G-protein-signaling. * indicate means significantly different. Circles indicate mean values ±SEM.

Activation of βγ proteins is mediated by regulators of G-protein-signaling or RGS. RGS proteins inhibit the activation of βγ proteins by *a* proteins (Abramow-Newerly et al., 2006). Inhibition of RGS proteins therefore potentiates MOR inhibition (Han et al., 2010). To determine whether RGS proteins are involved in opioid-induced respiratory rate depression, we directly inhibited RGS4 proteins, one of the most common RGS proteins in the nervous system, in the preBötC using the RGS4 inhibitor CCG50014 (Yoon et al., 2015). We microperfused CCG50014 (5 µM) in combination with DAMGO into the preBötC of adult anesthetized rats (Figure 4A) and compared respiratory changes with microperfusion of DAMGO alone. When the two conditions were compared (DAMGO alone *versus* DAMGO + CCG50014), DAMGO + CCG50014 produced significantly more pronounced respiratory rate depression than DAMGO alone (2-way ANOVAs, treatments *versus* conditions, *p = 0.002*, n = 5, Figure 4B). DAMGO alone (control condition: black line in Figure 4B) depressed respiratory rate by 13.6% (*p = 0.013*, n = 5), an effect not reversed by naloxone (*p = 0.093*, n = 5). In comparison, CCG50014 and DAMGO depressed respiratory rate by 37.6% (*p < 0.001*, n = 5). Addition of the MOR antagonist naloxone only reversed respiratory rate when compared to DAMGO + CCG50014 (*p < 0.001*, n = 5, Figure 4B). DAMGO did not significantly depress diaphragm amplitude (*p = 0.740*, Figure 4C).

**FIGURE 5:**
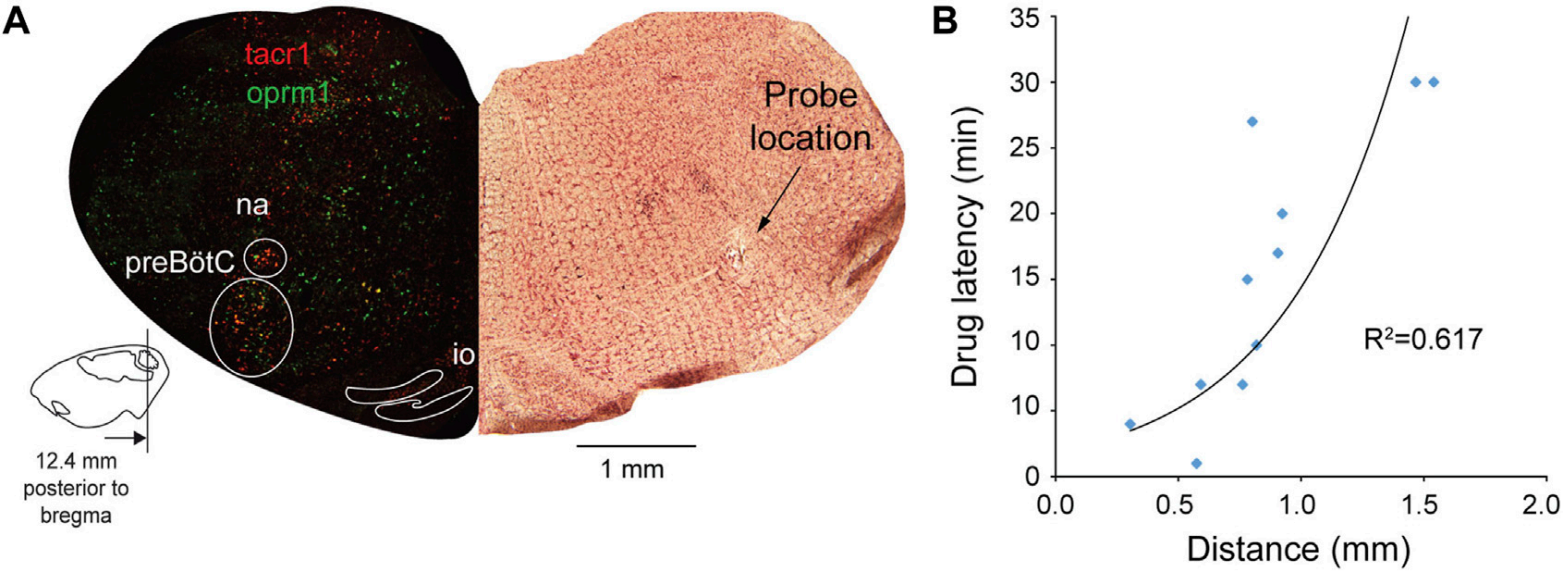
The site of action of DAMGO and CCG50014 overlaps with *Oprm1* and *Tacr1* expressing neurons. Using the same approach than in the gallein experiments, we determined the sites of drug perfusion and the latency for respiratory rate to decrease in response to DAMGO. Drugs were microperfused in the preBötC region as identified with expression of *Tacr1* (gene for neurokinin-1 receptors) **(A)**. A significant correlation was observed between distances from probe site to the centre of the preBötC and the latency for DAMGO to depress respiratory rate by 10% **(B)**. Na, nucleus ambiguus.

The fact that naloxone did not fully reverse respiratory rate depression by DAMGO + CCG50014 (Figure 4B, red line) suggests that CCG50014 decreased respiratory rate by a mechanism independent of MOR activation. To determine whether inhibition of RGS4 may directly change respiratory rate, we microperfused CCG50014 (5 µM) alone in the preBötC (Figure 4D). CCG50014 depressed respiratory rate by 20% (*p = 0.037*, n = 4), but did not change diaphragm amplitude (Figure 4E). In conclusion, inhibition of RGS4 proteins accentuated respiratory rate depression by DAMGO. However, this effect is due to the combination of inhibitory effects of CCG50014 and DAMGO and may not be due to the potentiation of MOR activation by RGS4 inhibition as proposed in this study. To confirm that perfusion of DAMGO and CCG50014 was performed in the region of the preBötC, we correlated the latency for DAMGO to depress respiratory rate with the location of the microperfusion sites (Figure 5A). As shown for gallein, microperfusion close to the preBötC center elicited a faster decrease in respiratory rate than microperfusion away from this region. A significant correlation was observed between latencies and distances from perfusion sites to the preBötC (R^2^ = 0.617, *p = 0.002*, Figure 5B), suggesting that DAMGO was acting directly in the preBötC region.

**FIGURE 6:**
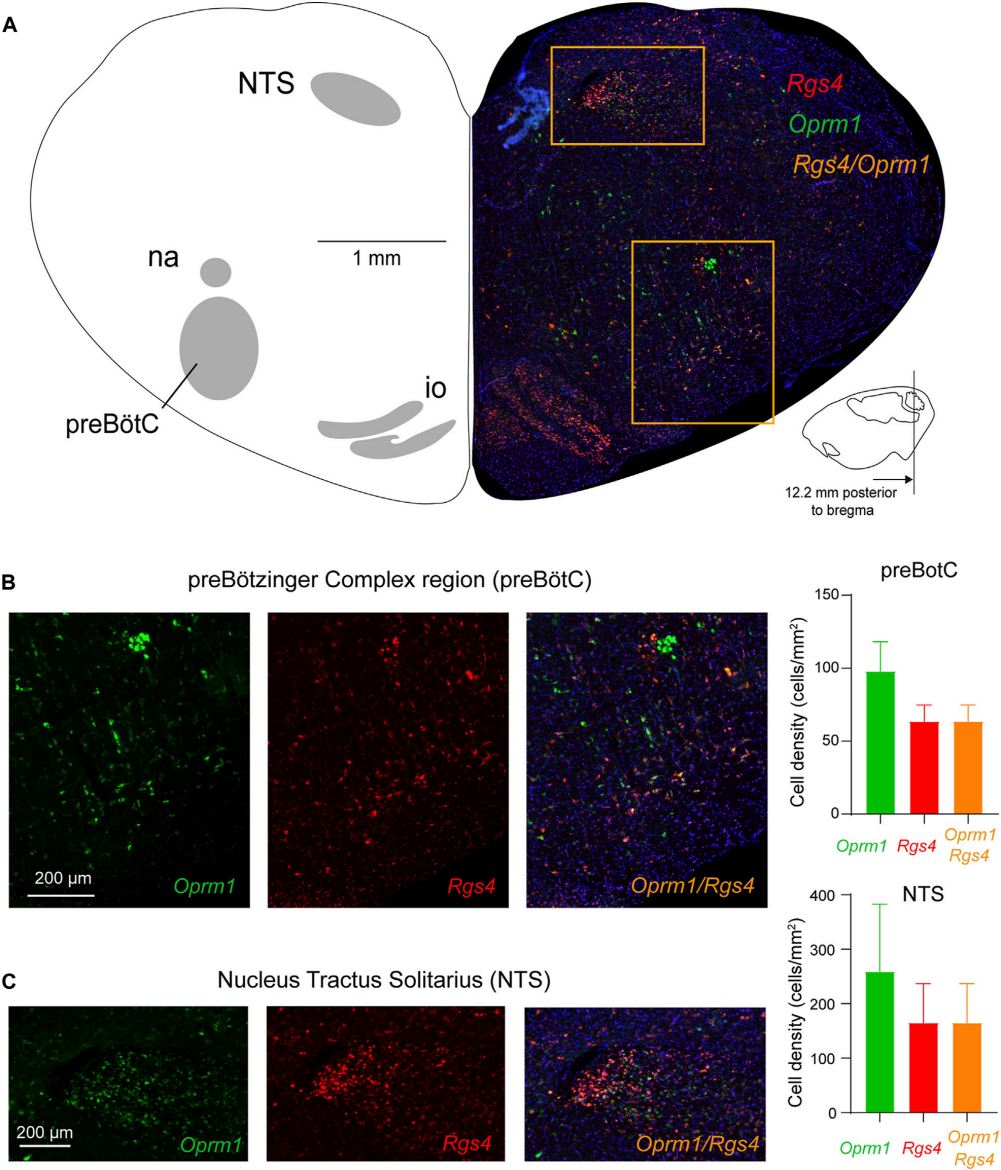
Expression of *Oprm1* and *Rgs4* mRNAs in the preBötC and NTS. **(A)** The preBötC and the nucleus tractus solitarius (NTS) expressed *Oprm1* (the gene coding for MORs, green) and *Rgs4* (the gene coding for regulators of G-protein signaling 4, red) mRNAs. **(B)** *Oprm1* and *Rgs4* mRNAs were expressed in the preBötC and most cells expressing *Oprm1* also co-expressed *Rgs4* (orange). The density of *Oprm1*-positive cells was moderate compared to the high density of *Oprm1* cells in the NTS **(C)**. In the NTS, cells expressed *Oprm1* as well as *Rgs4* with substantial co-expression. *Rgs4*, mRNA of regulator of G-protein signaling 4. *Oprm1*, Gene encoding MORs. NTS, nucleus tractus solitarius. na, nucleus ambiguus. io, inferior olive.

We then determined the expressions of *Rgs4* and *Oprm1* mRNA in the preBötC and NTS of adult rats (Figure 6) (Shi et al., 2021). *Oprm1* mRNA was found in the preBötC region and in the NTS as observed previously (Figure 1). Rgs4 was also observed in most cells of the preBötC and the NTS. Interestingly, most *Rgs4*-positive cells showed co-expression of *Oprm1* (Figure 6C) in the preBötC as well as the NTS. In fact, 60.2% ± 2.9% of *Oprm1*-positive cells co-expressed *Rgs4* in the preBötC and 60.1% ± 1.9% in the NTS. However, 33.4% ± 8.0% of *Oprm1*-positive cells did not co-express *Rgs4* in the preBötC and 33.4% ± 5.4% in the NTS. Interestingly, all *Rgs4*-positive cells co-expressed *Oprm1*. In summary, about 2/3 of *Oprm1*-expressing cells in the preBötC region co-expressed *Rgs4*, while about 1/3 of *Oprm1*-positive cells were exempt of *Rgs4*.

## Discussion

The preBötC constitutes a unique neural site in the medulla mediating respiratory depression by opioid drugs (Montandon et al., 2011; Varga et al., 2020). MOR activation of preBötC neurons, specifically neurons expressing neurokinin-1 receptors (Montandon et al., 2011), depresses respiratory rate in rats and mice (Montandon et al., 2016b). The downstream effectors regulating inhibition by MORs in the preBötC are not completely understood. We previously showed that GIRK channels regulate some components of respiratory depression by opioid drugs administered systematically or locally perfused in the preBötC (Montandon et al., 2016c). However, the role of the G-proteins activating GIRK channels following MOR activation is unknown. In this study, we showed that βγ proteins modulate respiratory rate depression by MOR activation. Blocking these proteins reversed the MOR inhibition in the preBötC and subsequent respiratory depression. To further investigate the roles of G-proteins in MOR inhibition, we modulated the regulator of G-protein signaling (RGS) and showed a limited role of RGS4 in modulating respiratory depression by activation of MORs, but an important role in the modulation of respiratory rhythm alone, suggesting that RGS4 proteins may regulate other G-protein-coupled receptors. Overall, our study supports that G-proteins may constitute a key-mechanism regulating MOR inhibition and respiratory depression and can provide new therapeutic targets to reverse or prevent respiratory depression by opioid drugs.

### MORs in the preBötC

The preBötC mediates opioid-induced respiratory depression as demonstrated by studies using mouse (Varga et al., 2020) and rat models (Montandon et al., 2011). MORs are found in the preBötC and are co-expressed with neurokinin-1 receptors (Gray et al., 1999). The MOR agonist DAMGO preferentially inhibits neurokinin-1 receptor-expressing preBötC neurons (Montandon et al., 2011) in rodents *in vitro*, therefore suggesting that neurokinin-1 receptor cells are keys in the regulation of opioid-induced respiratory depression. Microperfusion of the MOR agonist DAMGO in the region of the medulla expressing *tacr1* and *oprm1* significantly depressed respiratory rate. Using *in situ* hybridization to amplify multiple mRNA probes in the same section, we determined expression of *Oprm1* (MOR mRNA) and *Tacr1* (neurokinin-1 receptor mRNA) in key areas of the medulla. In the preBötC region, we showed a relatively high expression of *Oprm1* and co-expression of *Tacr1* in a subset of these cells. *Tacr1*-positive cells correspond to a sub-population of preBötC cells and are critical for respiratory rhythm (Gray et al., 2001). Although *Tacr1* expression is not found in all preBötC neurons, *Tacr1*-expressing cells are derived from Dbx1 progenitors, which are considered responsible for the generation of respiratory behaviors (Gray et al., 2010). Because dbx1 is not observed in adult mammals, *Tacr1* provides a relatively accurate indicator of preBötC cells in adult rodents. Here, we showed for the first time that *Tacr1* and *Oprm1* are co-expressed in the preBötC. Such co-expression is consistent with the fact that *Tacr1*-cells are preferentially inhibited by DAMGO *in vitro* (Montandon et al., 2011). In addition, microperfusion of DAMGO close to the preBötC, as identified with *Tacr1* expression, elicits a potent and fast respiratory depression, and the region were DAMGO acted also overlapped with *Oprm1* and *Tacr1*-positive neurons. In conclusion, our results confirm that DAMGO activates MORs and inhibits neurons in the preBötC and that a subset of *Oprm1*-positive cells also expressed *Tacr1*, therefore associating the functional effect of DAMGO and the co-expression of *Oprm1* in *Tacr1*-expressing preBötC cells.

### Gβγ subunit

The present study is the first to show that modulation of Gβγ subunit activity in the preBötC modulates respiratory rate depression by MOR ligands *in vivo.* Here we found that pharmacological inhibition of Gβγ subunit activity in preBötC neurons *in vivo* reversed respiratory rate depression by the MOR agonist DAMGO. Microperfusion of the MOR antagonist naloxone after perfusion of gallein did not significantly change respiratory rate. To demonstrate that gallein specifically blocked the effects on DAMGO and that Gβγ blocking in the preBötC did not affect respiratory rate by itself, we performed additional negative control. We perfused gallein alone and did not observe any change in respiratory rate despite perfusion in the preBötC according to brain histology. Intraperitoneal injection of gallein produced an increased morphine antinociceptive response using the warm-water tail-withdrawal assay, but it did not prevent respiratory depression by morphine (Hoot et al., 2013). The absence of effects of gallein can be explained by the fact that gallein is poorly soluble and may not easily cross the blood brain barrier, which may limit its availability in the brain when injected intraperitoneally. In fact, we used a relatively high concentration of gallein (5 mM) to elicit a sufficient reversal of DAMGO effect. On the other hand, the action of gallein on the antinociception produced by morphine may be due to its effects on peripheral nerve cells (Sun et al., 2019). By perfusing drugs directly in the medulla, our approach was not impeded by the brain blood barrier and can effectively block Gβγ subunits in the brain. Taken together, our data suggest that gallein reverses respiratory depression by locally applied MOR agonist to the brainstem circuits mediating respiratory depression by opioid drugs.

The Gβγ subunit is known to activate various MOR-induced signaling pathways, including inhibiting N-type calcium channels (Law et al., 2000) and activation of GIRK channels (Torrecilla et al., 2002). The role of calcium channel inhibition in respiratory depression by opioids is currently unknown. However, GIRK channel activation plays a significant role in mediating respiratory depression by MOR activation (Montandon et al., 2016b). Thus, the reversal of respiratory rate depression observed in our studies could be due to Gβγ subunit’s ability to activate GIRK channels, especially since we used a similar DAMGO concentration than in previous studies looking at the role of GIRK channels (Montandon et al., 2011; Montandon et al., 2016). However, it cannot be excluded that Gβγ subunits also regulate inhibition of calcium channels leading to neuronal inhibition. MORs may inhibit neuronal activity and lead to respiratory depression by inhibiting adenylyl cyclase through Gα subunit (Ballanyi et al., 1997). Elevation of cAMP levels by forskolin applied onto medullary neurons *in vitro* reversed fentanyl-induced respiratory depression (Ballanyi et al., 1997). Although this study showed that adenylyl cyclase activity can modulate fictive breathing *in vitro*, it has yet to be shown that *in vivo* modulation of adenylyl cyclase in the preBötC mediates respiratory depression by opioids. While these previous findings implicate molecular pathways that partially overlap with GIRK channel activation in respiratory depression by opioids, the reversal of respiratory rate depression by gallein administration suggests that Gβγ subunits are involved in MOR-induced suppression of neuronal activity in respiratory networks. Gallein inhibits Gβγ activity by competitively binding to the protein complex’s active site and blocking the binding of other molecules (Surve et al., 2014), including MOR ligands.

### Regulators of G-protein signaling

Understanding the distinct molecular pathways that underlie respiratory depression by activation of MORs is of great importance because opioids are widely used to manage pain (Volkow and McLellan, 2016) while presenting severe respiratory side-effects. Here, we showed that Gβγ proteins regulate respiratory depression following activation of MORs in the brainstem circuits mediating respiratory depression by opioid drugs. Gβγ proteins are mediated by regulators of G-protein signaling. Certain RGS subtypes are highly selective for specific opioid receptor subtypes; for example, RGS12 selectively acts on κ-opioid receptor signaling over other opioid receptor signaling pathways (Gross et al., 2019). RGS4 is able to modulate the analgesic effects of opioid drugs acting on MORs (Yoon et al., 2015), and these analgesic effects are largely mediated by MOR signaling (Kieffer, 1999). While RGS proteins, including RGS4, have been shown to modulate opioid analgesia (Yoon et al., 2015), reward (Kim et al., 2018), and tolerance pathways (Stewart et al., 2015), RGS role in respiratory depression was yet to be characterized. To demonstrate the potential role of RGS4 in respiratory depression by MOR activation, we performed unilateral inhibition of RGS4 in the area of the preBötC *in vivo*. RGS4 inhibition with the RGS4 inhibitor CCG 50014 did not significantly potentiate the respiratory rate depression induced by DAMGO. Interestingly, although large fraction of *Oprm1*-positive cells also co-expressed *Rgs4* mRNA, about 30% of *Oprm1*-positive cells did not express *Rgs4*. In rats studied *in vivo*, administration of the RGS4 inhibitor CCG 50014 was able to strongly potentiate the analgesic effects of opioids and decrease the response to nociceptive stimuli by greater than 50% (Yoon et al., 2015). In fact, the potency of DAMGO was increased 10-fold in the formalin test in animals lacking RGS4 compared to their wild-type controls (Yoon et al., 2015), and intrathecal administration of the small molecule RGS4 inhibitor CCG-50014 to wild-type mice produced dose-dependent antinociception (Blazer et al., 2010) that was blocked by the opioid antagonist naloxone, as well as enhancing the action of DAMGO (Yoon et al., 2015). RGS4 are widely distributed in the central nervous system where it regulates MOR activation (Traynor, 2012). In neuroblastoma cells expressing endogenously RGS4 and MOR, knockdown of RGS4 does not affect the action of morphine on MOR, suggesting that the ability of RGS4 to regulate MOR may be determined by the cell type and/or agonist. Importantly, RGS9 plays a major role in regulation MOR inhibition, but there are no drugs that can directly block RGS9 (Zachariou et al., 2003). Overall, RGS4 does not mediate respiratory depression by MOR, but may be involved in MOR inhibition of other circuits such as neural circuits of nociception.

RGS4 inhibition alone, in the absence of opioids, caused a significant decrease in respiratory rate. Here, we showed that RGS4 is endogenously expressed in the preBötC and it has been shown that it functions as a GAP in other neuronal populations (Soundararajan et al., 2008). For instance, RGS4 also inhibits somatostatin signaling *in vitro* (Huang et al., 1997) and the neuropeptide somatostatin modulates respiratory rate in the preBötC (Montandon et al., 2016a). Somatostatin and its cognate receptors are expressed in the preBötC (Stornetta et al., 2003) and are necessary for generating rhythmic breathing (Gray et al., 2001; Tan et al., 2008). Somatostatin is known to decrease rhythmic breathing by acting on somatostatin 2 receptors (Gray et al., 2010), through GIRK channels (Montandon et al., 2016a), therefore suggesting that RGS4 inhibition may potentiate the effects of endogenous somatostatin on respiratory rhythm, but not the inhibitory effects of MOR. On postsynaptic membranes, RGS proteins also modulate fact-acting neurotransmission mediated by ligand-gated ion channels, including glutamate and γ-aminobutyric acid (GABA) receptors (Gerber et al., 2016), two of which are also involved in respiratory rhythm generation (Del Negro et al., 2018).

The role of G-protein signaling in MOR inhibition of respiratory circuits has been a controversial topic over the last 2 decades. It was originally suggested that respiratory depression was regulated by the adapter protein *ß*-arrestin which forms complexes with GPCR (Raehal et al., 2005). It was suggested respiratory depression by MOR was due to *ß*-arrestin recruitment, while opioid analgesia was mediated by G-protein signaling (Bohn et al., 1999). These results led to the concept of biased agonism, where biased agonists may have differential effects on nociception and breathing due to their ability to trigger G-protein signaling or *ß*-arrestin recruitment (Mores et al., 2019). However, it was recently demonstrated that MOR inhibition of respiratory circuits did not depend upon *ß*-arrestin recruitment (Kliewer et al., 2019; Kliewer et al., 2020). The importance of G-protein signaling in regulating MOR inhibition of respiratory circuits is further supported by the role of GIRK channels (Montandon et al., 2016b) and Gβγ dimers in opioid-induced respiratory depression demonstrated in the present study.

### Caveats and limitations of the study

In this study, we used the volatile anesthetic isoflurane which acts on GABA_A_ receptors and significantly alters respiratory rate (Wilkerson et al., 2016). It is plausible that it also affects the role of Gβγ subunits and RGS in the regulation of breathing. However, this approach allows more stable respiration over time compared to other anesthetics. In addition, we did not measure blood gases which may be significantly affected by the combination of respiratory depression by DAMGO and the volatile anesthetic isoflurane. However, we supplemented inspired air with 50% oxygen to ensure stable oxygen levels throughout the experiments (Nagappa et al., 2017). Importantly, the findings obtained under anesthetized conditions with microperfusion of DAMGO in the preBötC have been replicated in freely-behaving, non-anesthetized experiments (Dahan et al., 2010; Nagappa et al., 2017), which suggest that results during anesthesia may be replicated in freely-behaving conditions. Importantly, experiments under anesthetized conditions eliminate the confounding factor of changing sleep-wake states during the experiment and enable more consistent respiratory depression by opioid ligands (Montandon et al., 2016b; Nagappa et al., 2017).

In conclusion, Gβγ dimers are essential G-proteins involved in MOR inhibition of respiratory circuits and may be key-players in respiratory depression by opioid drugs. Following MOR activation by opioid ligands, Gα is activated and releases Gβγ proteins, which directly binds to and activates GIRK channels (Abramow-Newerly et al., 2006), consistent with the role of GIRK channels in respiratory depression by opioids (Montandon et al., 2016b). Importantly, GIRK channels hyperpolarize the neuron and dampen the overall capacity of the postsynaptic signaling to potentiate. The rate at which GIRK channels close following ligand removal is regulated by RGS proteins, which modulate the capacity of the postsynaptic membrane to depolarize following MOR activation. In conclusion, RGS proteins may be involved in depotentiation following MOR activation rather than hyperpolarization which would explain the lack of role of RGS in respiratory depression by the MOR ligand. The functional role of Gβγ proteins in the regulation of respiratory depression by opioid drugs suggests that Gβγ proteins may serve as molecular targets to develop therapies that could reverse respiratory depression following opioid overdose, especially in the context of highly potent fentanyl analogs for which the opioid overdose antidote naloxone is not effective (Pergolizzi, 2021).

## Data Availability

The raw data supporting the conclusion of this article will be made available by the authors, without undue reservation.
