# Supplementary material for: βγ G-proteins, but not regulators of G-protein signaling 4, modulate opioid-induced respiratory rate depression
Source: Front Physiol. 2023 Apr 6;14:1043581. doi: 10.3389/fphys.2023.1043581 (PMC10117644; doi:10.3389/fphys.2023.1043581)
Supplement: Supplementary file 1 [file DataSheet1.pdf]

Animal #1

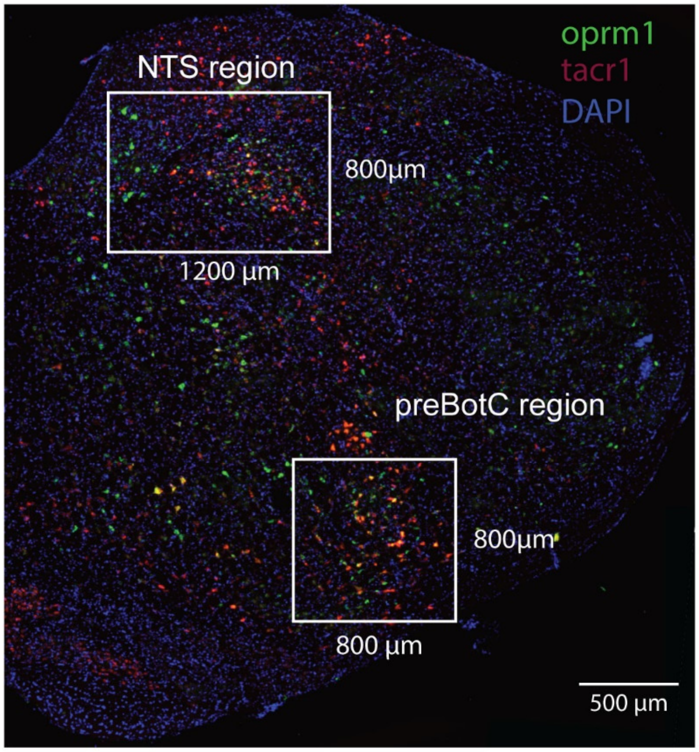

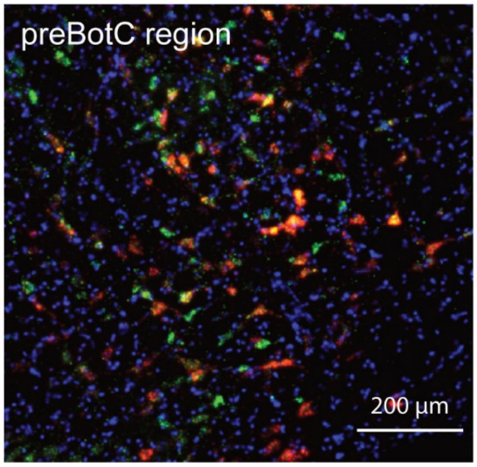

preBotC

oprm1-positive cells = 53

tacr1-positive cells = 63

tacr1/oprm1-positive cells = 27

NTS

oprm1-positive cells = 60

tacr1-positive cells = 21

tacr1/oprm1-positive cells = 15

Supplementary Figure 1a. In-situ hybridization for Oprm1 and Tacr1 and cell counting for the preBötzinger Complex and the nucleus tractus solitarius.

Animal #2

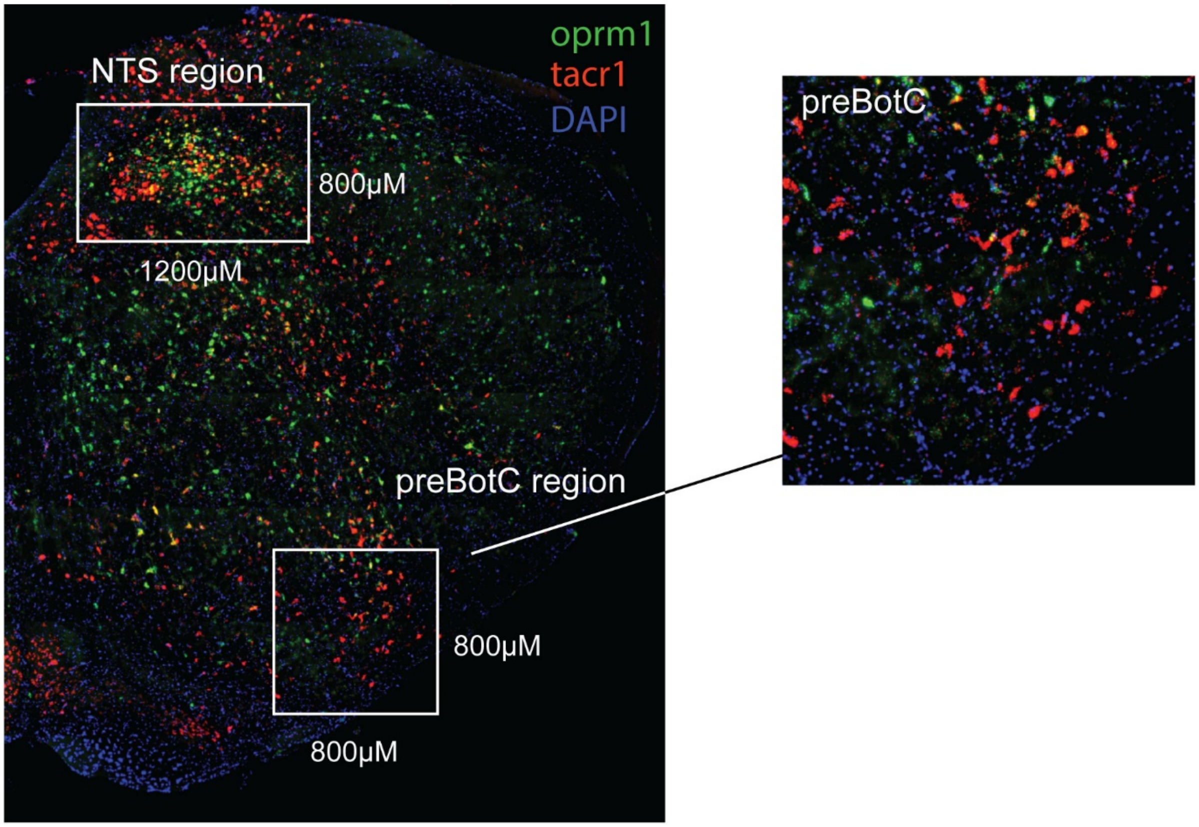

preBotC

oprm1-positive cells = 22

tacr1-positive cells = 50

tacr1/oprm1-positive cells = 17

NTS

oprm1-positive cells = 82

tacr1-positive cells = 66

tacr1/oprm1-positive cells = 51

Supplementary Figure 1b. In-situ hybridization for Oprm1 and Tacr1 and cell counting for the preBötzinger Complex and the nucleus tractus solitarius.

Animal #3

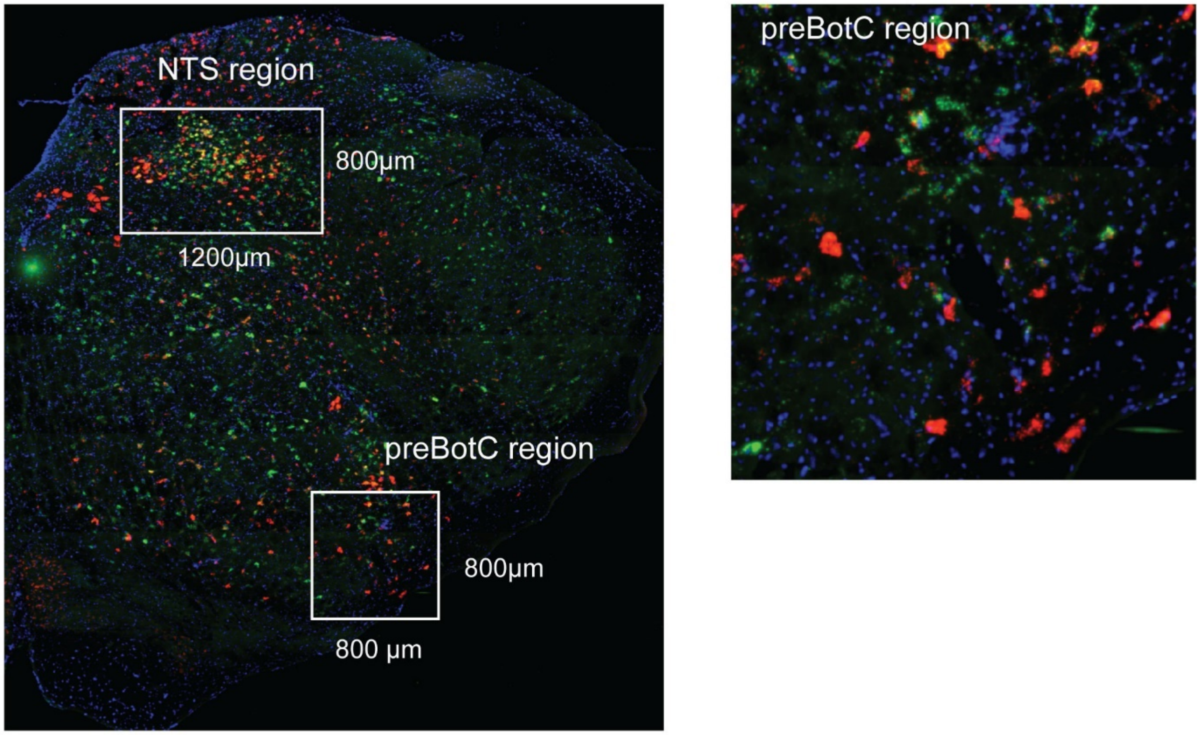

preBotC

oprm1-positive cells = 15
tacr1-positive cells = 24
tacr1/oprm1-positive cells = 9

NTS

oprm1-positive cells = 110
tacr1-positive cells = 68
tacr1/oprm1-positive cells = 54

Supplementary Figure 1c. In-situ hybridization for Oprm1 and Tacr1 and cell counting for the preBötzinger Complex and the nucleus tractus solitarius.

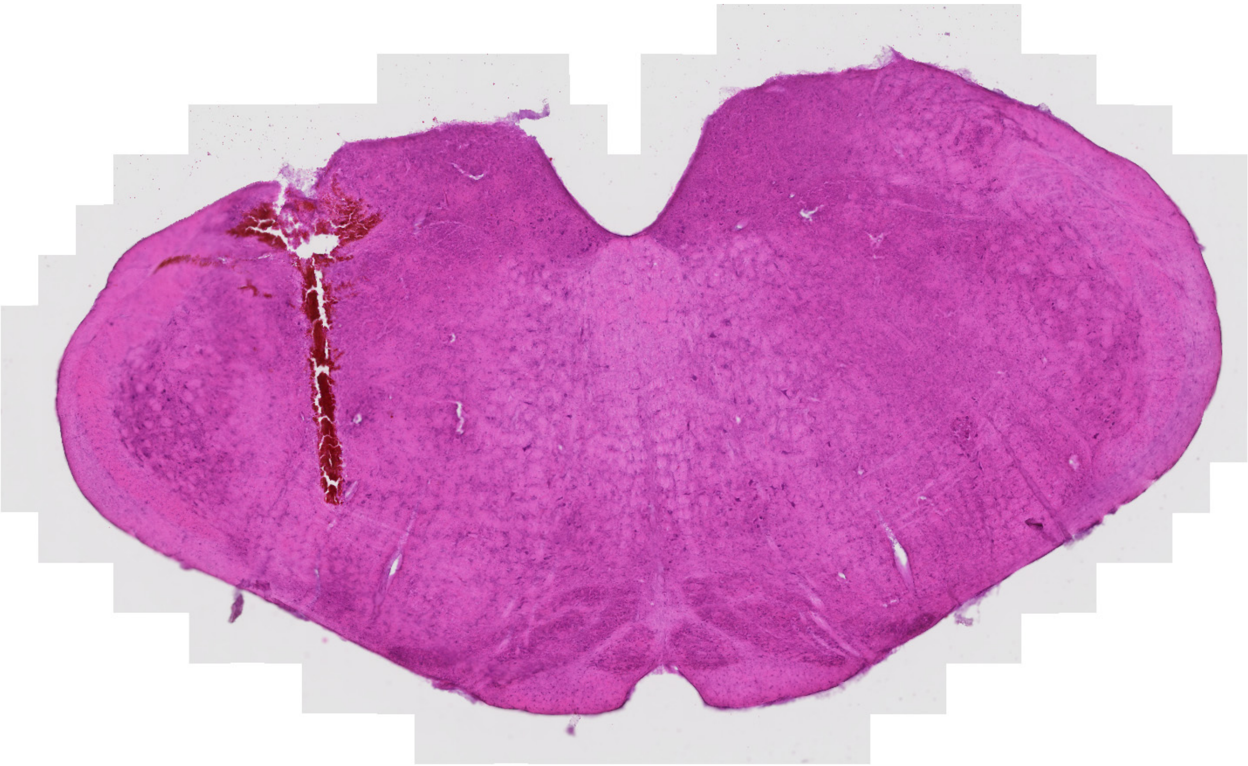

Supplementary Figure 3. Histology of the probe location for positive control of Figure 3. Medullary section 12.4 mm caudal to Bregma. Note that the probe is located just below the nucleus ambiguus.

Animal #11

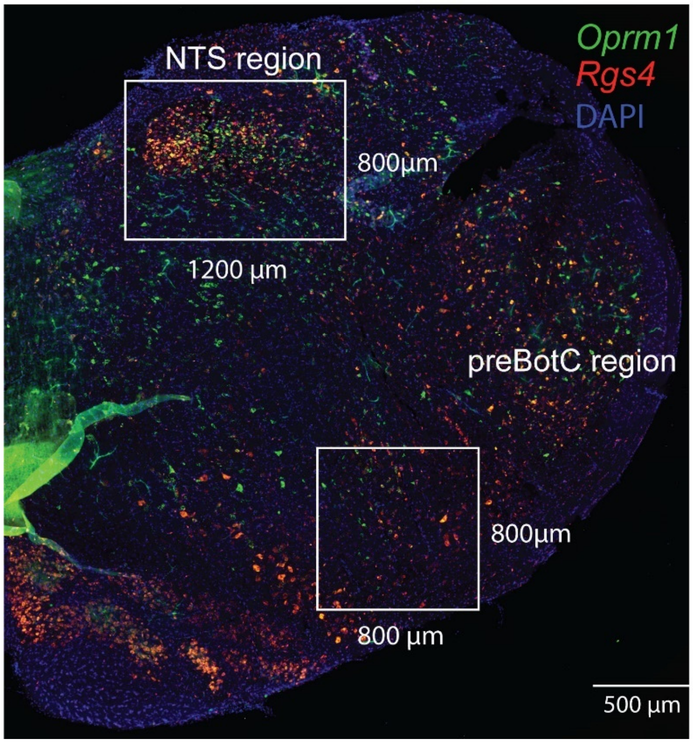

preBotC

oprm1-positive cells only = 25

Rgs4-positive cells only = 0

Rgs4/oprm1-positive cells = 53

NTS

oprm1-positive cells only = 189

Rgs4-positive cells only = 0

Rgs4/oprm1-positive cells = 296

Supplementary Figure 6a. In-situ hybridization for *Oprm1* and *Rgs4* and cell counting for the preBötzinger Complex and the nucleus tractus solitarius.

Animal #4

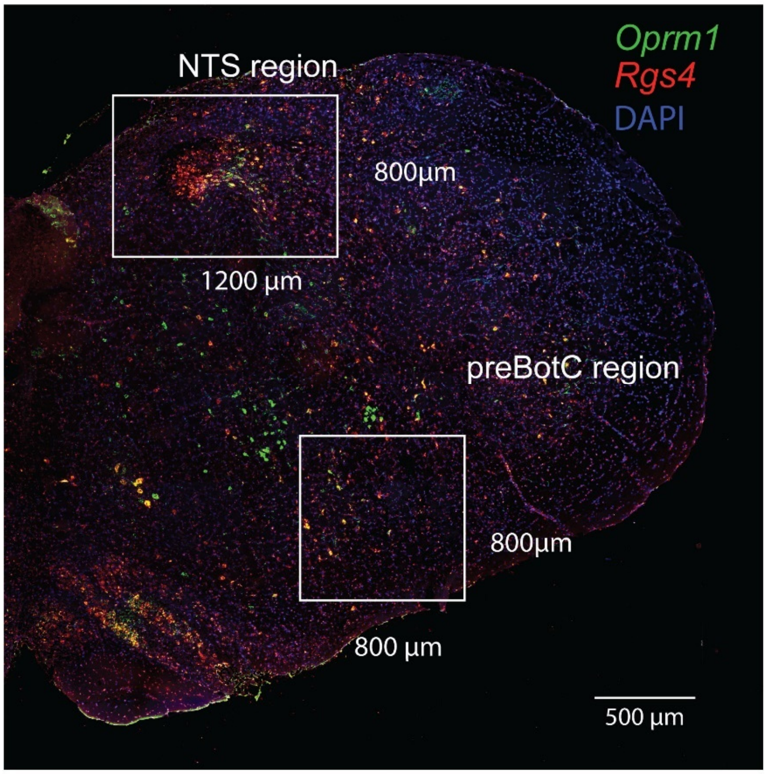

preBotC

oprm1-positive cells only = 9

Rgs4-positive cells only = 0

Rgs4/oprm1-positive cells = 28

NTS

oprm1-positive cells only = 28

Rgs4-positive cells only = 0

Rgs4/oprm1-positive cells = 79

Supplementary Figure 6b. In-situ hybridization for *Oprm1* and *Rgs4* and cell counting for the preBötzinger Complex and the nucleus tractus solitarius.

Animal #13

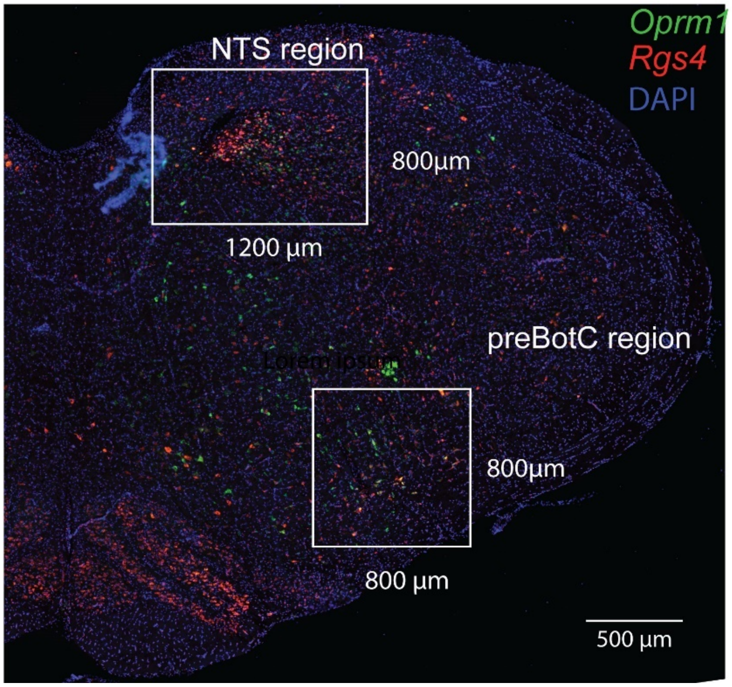

preBotC

oprm1-positive cells only = 32

Rgs4-positive cells only = 0

Rgs4/oprm1-positive cells = 41

NTS

oprm1-positive cells only = 54

Rgs4-positive cells only = 0

Rgs4/oprm1-positive cells = 100

Supplementary Figure 6c. In-situ hybridization for *Oprm1* and *Rgs4* and cell counting for the preBötzinger Complex and the nucleus tractus solitarius.
